# Optimal Clinical Time for Reliable Measurement of Transcutaneous CO_2_ with Ear Probes: Counterbalancing Overshoot and the Vasodilatation Effect

**DOI:** 10.3390/s100100491

**Published:** 2010-01-11

**Authors:** Christian Domingo, Elisa Canturri, Amalia Moreno, Humildad Espuelas, Laura Vigil, Manel Luján

**Affiliations:** 1 Pneumology Service, Hospital de Sabadell, Corporació Parc Taulí, Sabadell 08208, Spain; E-Mails: ecanturri@saas.ad (E.C.); amoreno@tauli.cat (A.M.); hespuelas@tauli.cat (H.E.); lvigil@tauli.cat (L.V.); mlujan@tauli.cat (M.L.); 2 Department of Medicine, Autonomous University of Barcelona (UAB), Cerdanyola del Valles, Barcelona 08193, Spain

**Keywords:** transcutaneous CO_2_, optimal reading time, overshoot phenomenon

## Abstract

**OBJECTIVES:**

To determine the optimal clinical reading time for the transcutaneous measurement of oxygen saturation (SpO_2_) and transcutaneous CO_2_ (TcPCO_2_) in awake spontaneously breathing individuals, considering the overshoot phenomenon (transient overestimation of arterial PaCO_2_).

**EXPERIMENTAL SECTION:**

Observational study of 91 (75 men) individuals undergoing forced spirometry, measurement of SpO_2_ and TcPCO_2_ with the SenTec monitor every two minutes until minute 20 and arterial blood gas (ABG) analysis. Overshoot severity: (a) mild (0.1–1.9 mm Hg); (b) moderate (2–4.9 mm Hg); (c) severe: (>5 mm Hg). The mean difference was calculated for SpO_2_ and TcPCO_2_ and arterial values of PaCO_2_ and SpO_2_. The intraclass correlation coefficient (ICC) between monitor readings and blood values was calculated as a measure of agreement.

**RESULTS:**

The mean age was 63.1 ± 11.8 years. Spirometric values: FVC: 75.4 ± 6.2%; FEV_1_: 72.9 ± 23.9%; FEV_1_/FVC: 70 ± 15.5%. ABG: PaO_2_: 82.6 ± 13.2; PaCO_2_: 39.9.1 ± 4.8 mmHg; SaO_2_: 95.3 ± 4.4%. Overshoot analysis: overshoot was mild in 33 (36.3%) patients, moderate in 20 (22%) and severe in nine (10%); no overshoot was observed in 29 (31%) patients. The lowest mean differences between arterial blood gas and TcPCO_2_ was −0.57 mmHg at minute 10, although the highest ICC was obtained at minutes 12 and 14 (>0.8). The overshoot lost its influence after minute 12. For SpO_2_, measurements were reliable at minute 2.

**CONCLUSIONS:**

The optimal clinical reading measurement recommended for the ear lobe TcPCO_2_ measurement ranges between minute 12 and 14. The SpO_2_ measurement can be performed at minute 2.

## 1. Introduction

During the last 50 years, there has been a growing interest in arterial blood gas measurements for calculating PaO_2_ [1,2] and PaCO_2_ [3,4]. Non-invasive measurement of both PaCO_2_ and PaO_2_ has presented major technical and practical problems [2], but pulse oximetry, introduced in 1985, proved to be effective for the non-invasive assessment of oxygenation during sleep [1] and exercise [2]. No techniques were available for non-invasive continuous monitoring of PaCO_2_.

At the turn of the century, Rohling and Biro [5] and Tschupp and Fanconi [6] published the first descriptions of a monitor that combined the elements of an optical pulse oximetry sensor with a Severinghaus-type PaCO_2_ sensor, which has been used in several clinical situations [7]. One of the key points that remain to be determined is the time at which the monitor offers the most reliable measurement.

In a previous study, we validated the combined oximetry and transcutaneous CO_2_ (TcPCO_2_) ear-lobe monitor (Sentec Corporation, Inc) designed for SpO_2_ and TcPCO_2_ measurements [3]. In that study we established empirically that the monitor needed a maximum stabilization time of 17 minutes in order to perform the reading of the PaCO_2_ value. Considering that SpO_2_ reading is much faster than PaCO_2_, the purpose of this study was to determine the optimal time for the TcPCO_2_ reading of the Sentec monitor in a cohort of clinically stable individuals including patients and normal controls.

## 2. Experimental Section

Based on our previous results, we hypothesized that the stabilization time at which the Sentec monitor was reliable for SpO_2_ and PaCO_2_ measurements would be <17 minutes [3].

### Patients

Inclusion criteria: (a) a consecutive Caucasian adult population referred to the respiratory function testing clinic of our hospital for forced spirometry and arterial blood gas analysis.

Exclusion criteria: (a) active smoking habit; b) jaundice; (c) recent hospital admission (<3 months); d) clinical hypotension; (e) refusal to participate. Since our previous validation study had shown that the over- and underestimation by the monitor was not clinically relevant [3], patients were not stratified and grouped according to pulmonary function, even though this variable was measured.

### Methods

The study was observational and cross-sectional. All patients underwent forced spirometry (System 1070, Series 2E/1085, MedGraphics, St. Paul, MI, USA) on the day of the study. Spirometry was carried out in accordance with the guidelines of the Spanish Society for Pulmonology and Thoracic Surgery (SEPAR) [8]. Reference values used were those published for a Mediterranean population [9]. SpO_2_ and TcPCO_2_ were then measured with the V-SignTM combined monitor (SenTec Inc, Therwil, Switzerland). A screen reading of the Sentec monitor for SpO_2_ and TcCO_2_ was performed every two minutes until minute 20. We defined the overshoot phenomenon as the transient overestimation of arterial PaCO_2_ by the monitor at least during two consecutive readings (2 minutes). The severity of overshoot was classified as follows: (a) mild: transient overestimation of arterial PaCO_2_ between 0.1 and 1.9 mm Hg; (b) moderate: transient overestimation of arterial PaCO_2_ between 2 and 4.9 mm Hg; c) severe: transient overestimation of arterial PaCO_2_ > 5 mm Hg.

After the stabilization period and once screen monitor readings were completed, arterial blood gas (ABG) analysis was performed in accordance with SEPAR guidelines [10] which include the use of subcutaneous anaesthesia before puncture. The sample for arterial blood gas analysis was processed in two analyzers and hemoximeters belonging to the respiratory function testing laboratory (Radiometer ABL 500 series, Copenhagen, Denmark; Radiometer OSM3 hemoximeter) and immediately afterwards using the instruments at our intensive care unit (ICU) (Radiometer ABL 700 series; Radiometer OSM3 hemoximeter). Although the two laboratories are on the same floor and close to one another, the blood samples were transported between the two sites on ice. At least two readings were taken with each analyzer for each blood sample. The optimal value was always taken (highest PaO_2_ and lowest PaCO_2_) when the values differed by less than 1 mm Hg; otherwise a third reading was recorded. The arithmetic mean of the readings from the two analyzers was calculated and the result was taken as the reference value from the arterial blood gas analysis. The body temperature of the patient was specified on analysis of the blood sample.

### Statistical Analysis

Anthropometric, spirometric and ABG data were expressed as means (SD). ABG values processed in the respiratory function testing unit were compared with the ICU values by calculating the mean differences for PaCO_2_ and oxygen saturation (SpO_2_). The Pearson correlation coefficients (*r*) between PaCO_2_ and TcPCO_2_ and between SaO_2_ and SpO_2_ were determined. Finally, a concordance study was performed by calculating the single measure intraclass correlation coefficient (ICC). An ICC > 0.71 indicates a good concordance between the two measures, and a very good concordance when it is >0.9. The ANOVA test was used to compare TcPCO_2_ at different measurement times. The study was approved by the ethics committee of our hospital.

## 3. Results

Ninety-one patients (75 men) were included in the study. Demographic data, pulmonary function testing (PFTs) and ABG values are shown in Table 1.

The heterogeneity of the cohort of patients reflects the presence of normal controls as well as COPD patients with different degrees of airway obstruction.

As Table 2 shows, the mean differences between arterial and transcutaneous CO_2_ in the first two readings were substantial (4 min). Mean differences were below 1 mm Hg after minute 6, but the maximum agreement with an ICC > 0.8 was achieved after minute 12 of measurement. These agreement values did not worsen in the following minutes. Regarding oximetry saturation values, the agreement between oximeter amd arterial oxygen saturation was very good even in the first reading (minute 2: Table 3).

**Figure 1.**
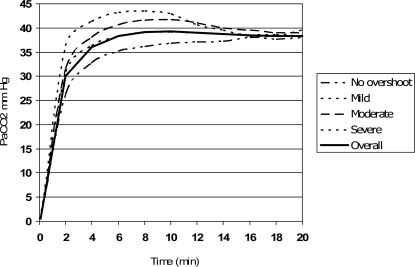
The TcPCO_2_ values tend to converge after minute 10. At minutes 12 and 14, the overshoot phenomenon seems to disappear.

Overshoot analysis: overshoot was mild in 33 (36.3%) patients, moderate in 20 (22%) and severe in 9 (10%); no overshoot was observed in 29 (31%) patients. No association was found between demographics or baseline levels of PaCO_2_ and overshoot. As Table 4 shows, early readings (first 10 minutes) showed statistical significant differences in patients with moderate-to severe overshoot and those without. These differences disappear after minute 12, at which point ICC was >0.8 (Table 2). Figure 1 shows the relationship between the different groups at each time measurement.

When patients with severe overshoot are excluded from the analysis, readings before minute 14 improved slightly, with ICC values of 0.240 (m2), 0.521 (m4) 0.655 (m6), 0.736 (m8), 0.782 (m10), 0.804 (m12) and 0.838 (m14). After minute 14 no relevant differences were observed.

## 4. Discussion and Conclusions

The stabilization time had been established empirically in a previous study [3] on the basis of the opinion of the nurse who considered the correct reading to be at about 14 ± 3 minutes. The next step was to establish the optimal (shortest and reliable) screen monitor reading time for the TcPCO_2_ and for the SpO_2_. We decided to use an interval of two minutes between measurements since previous experience had shown that this was the time taken by the monitor to detect the occurrence of an event [11]. As in our previous study [3] we took readings until 20 minutes after attaching the sensor to the ear lobe. None of our patients had dark skin, were smokers or had jaundice, and so the possible effects of these factors on the results of pulse oximetry could be ruled out. Arterial blood gas analysis was performed in accordance with SEPAR guidelines [10]. To avoid systematic calibration errors of the analyzers, we processed our samples in two different analyzers and took the mean of the values obtained as the reference arterial blood gas value, as in our previous study [3].

As Table 2 shows, the mean differences between arterial and transcutaneous CO_2_ are substantial in the first two readings (4 min). By minute 6 the mean differences fell below 1 mm Hg, but the maximum agreement with a ICC > 0.8 was achieved after measurement at minute 12; the fact that these agreement values did not worsen in the following readings suggest that there was no drift during these first 20 minutes of monitoring. Regarding oximetry saturation values, the agreement between oximeter and arterial oxygen saturation was excellent from minute 2 onwards.

Two opposite phenomena may interfere with TcPCO_2_ readings during the first minutes of monitoring. First, the arterialization of capillary blood due to vasodilatation lasts several minutes. Second, the metabolism of the cells on the skin surface is increased by the application of heat, which increases the release of CO_2_ from the cells, thus causing a transient overestimation of true PaCO_2_ (the “overshoot phenomenon”). It is believed that the overshoot usually precedes the complete vasodilatation of subdermal tissue. Kagawa *et al.* [12] confirmed the overshoot phenomenon during the early stages of TcPCO_2_ measurement. Hirabashi *et al.* [13] examined a method for avoiding overshoot in eight adult patients under general anaesthesia, and found that their device prevented it if the probe was heated to 45 °C rather than to 42°. Since overshoot is temperature-dependent, it might be thought that heating patients’ tissue to a lower temperature would reduce the phenomenon, but Hirabashi *et al.* observed the opposite: by heating the tissue to a higher temperature in the early stages, overshoot and vasodilatation tended to coincide in time, thus minimizing the problem. We should stress that Hirabashi’s patients were under general anaesthesia, whereas our patients were awake and breathing spontaneously.

The device we tested heats the tissue to 41 °C. Under these conditions, we observed severe overshoot (>5 mm Hg) in 10% of patients. Interestingly, mean TcPCO_2_ between patients with or without overshoot (Table 4) was not statistically different after minute 12, suggesting that this phenomenon interferes with readings only in the first 12 minutes. Moreover, when patients with severe overshoot were excluded from the analysis, the intraclass correlation coefficient after minute 12 did not change. Overshoot seems to be an individual phenomenon that is unpredictable before monitoring. High early TcPCO_2_ readings may alert the clinician to its presence (Table 4) although the interpretation of this value depends on the patient’s baseline PaCO_2_. We were unable to identify a cut-off point that was sufficiently sensitive and specific for use in clinical practice.

Measurements are a fundamental part of both clinical practice and investigation. Repeated measurements of the same variable in the same subject do not usually yield the same value. This may be due to natural variations in the subject, variations in the measurement process, or both [14]. Therefore, statistical methods to calculate the error are important. As previously discussed [3], although the Pearson correlation coefficient is useful as a first approach, it measures the strength of linear association of two variables and indicates how the values of one vary as a function of the other; so it only allows us to rule out the negative hypothesis, that is, that there is no linear association between the two measurements (the reference measurement and the measurement for validation). A strong correlation, however, does not necessarily mean that the two measurements agree [15–16]. To validate a technique, we must not only show that the two variables vary in the same direction but that they do so in the same way.

The reliability of a device or method of analysis should thus be evaluated with tests of agreement. We used the ICC in our study because a number of authors have suggested that it provides a measure of agreement for continuous variables. This coefficient is not subject to the problems inherent in the linear correlation coefficient: it does not depend on the range of values of the sample, and the result is unaffected by the order in which the readings are presented or by inter-subject variability, although changes in measurement scales do have an effect [3]. It is also an excellent indicator of reliability of the measurement [3].

Besides the mathematical results, in clinical practice we have to take the decision which is most clinically useful, and which does not always coincide with the most exact measurement. Kagawa and Severinghaus underlined [17] that the best moment to take a TcPCO_2_ measurement was at minute 30 after placing the ear probe, but in many clinical situations, the prompt availability of the result is at least as important as the value itself – for instance, in the emergency room or in ambulatory care when visits tend to be scheduled very frequently. In these cases, shortening the time required to obtain the measurement is crucial. We observed that, at minutes 8 and 10, the mean differences between arterial and transcutaneous CO_2_ values were clinically insignificant and that the intraclass correlation coefficient was very good, showing that in most measurements at this time the correlation between the two techniques would be reliable. Taking into consideration the overshoot phenomenon, what happens in these early readings is that some values compensate the others and make the mean differences for the whole group very low. Beyond minute 12, although the mean differences are not lower, the ICC is higher, thus illustrating the compensation of the overshoot phenomenon by vasodilatation. Figure 1 shows that at minute 12 readings tend to converge, thus minimizing the error.

Although direct measurement of PaCO_2_ remains the gold standard, it provides only a single measurement of what is often a rapidly changing and evolving clinical picture. As a result, the clinical need remains for a means to continuously monitor PaCO_2_ without the need for repeated blood gas analysis. Transcutaneous CO_2_ monitoring can be applied in situations [18,19] that generally preclude end tidal CO_2_ monitoring such as high frequency ventilation, apnea testing, and noninvasive ventilation [20]. Moreover, the technique has shown to be more reliable than capnometry [4,21].

This study has several limitations. First, we had only one value for the PaCO_2_ and we assumed that it did not change throughout the study. The second limitation is that our patients were awake and spontaneously breathing, which makes it difficult to compare our results with other studies with anaesthetized patients. Finally, our monitor is different from others frequently used in other papers. Therefore, our conclusions apply to the monitor and circumstances described and may not be applicable to all situations.

In our setting, ambulatory care of COPD patients [22] in specialized units [23] is very frequent. Repeated arterial punctures for the measurement of ABG have remained one of the most important patient concerns. A solution for oxygenation measurement was found many years ago with arterial pulse-oximetry, but the approach to PaCO_2_ with non-invasive methods has remained a problem until recently. Since a single measurement is needed for every outpatient, it is crucial to establish the optimal time at which to perform the reading. Transcutaneous carbon dioxide monitoring may be a useful adjunct in various clinical scenarios and may help to improve control and therapy in many patients in the coming years [24].

## Figures and Tables

**Table 1.** Demographic data and pulmonary function testing and arterial blood gas values (mean ± SD) of 91 patients included in the study.

**Variable**	**Mean ± SD**	**Range**

**Age (years)**	63.1 ± 11.8	37–86
**FEV_1_ (l)**	2.21 ± 0.87	0.51–4.33
**FEV_1_%**	72.92 ± 23.9	17–124
**FVC (l)**	3.13 ± 0.92	1.23–5.14
**FVC %**	75.4 ± 16.2	37–107
**FEV_1_/FVC**	70 ± 15.6	24–88
**pH**	7.41 ± 0.02	7.35–7.48
**PaCO_2_ (mm Hg)**	39.91 ± 4.85	29–55
**PaO_2_ (mm Hg)**	82.6 ± 13.23	49.2–126
**HCO_3_ (mmol/L)**	25.5 ± 2.14	19–32
**SaO_2_ (%)**	95.7 ± 2.30	85–99
**Hb (g/L)**	14.26 ± 1.79	9.2–19

**Table 2.** Values of TcPCO_2_ readings, mean differences between PaCO_2_ and TcPCO_2_, correlation and intraclass correlation coefficient for individual measures each 2 minutes.

	**2’**	**4’**	**6’**	**8’**	**10’**	**12’**	**14’**	**16’**	**18’**	**20’**

**Mean TcPCO_2_ (SD)**	30.1 (7.5)	36.04 (6.7)	38 (7.5)	39.2 (6.2)	39.3 (5.8)	39 (5.6)	38.7 (5.5)	38.6 (5.3)	38.2 (5.3)	38.15 (5.1)
**Mean PaCO_2_-TcPCO_2_ (SD)**	9.6 (7.6)	3.8 (5.8)	1.8 (6)	0.7 (4.2)	0.57 (3.5)	0.87 (3.3)	1.14 (3)	1.28 (2.9	1.6 (3)	1.7 (2.8)
**95 % CI**	8.0–11.2	2.5–5	0.5–3	−0.2–1.5	−0.17–1.32	0.17–1.56	0.5–1.8	0.6–1.9	0.9–2.2	1.1–2.3
**Pearson correlation coefficients (*r*)**	0.292	0.549	0.594	0.741	0.791	0.810	0.834	0.834	0.822	0.838
**ICC**	0.267	0.519	0.54	0.718	0.777	0.801	0.827	0.830	0.819	0.836

The upper row shows the time in minutes. TcPCO_2_: Transcutaneous CO_2_. SD: standard deviation. CI: confidence interval. ICC: intraclass correlation coefficient for individual measures

**Table 3.** Values of SpO_2_ readings, mean differences between SaO_2_ and SpO_2_, correlation and intraclass correlation coefficient for individual measures each 2 minutes.

	**2’**	**4’**	**6’**	**8’**	**10’**	**12’**	**14’**	**16’**	**18’**	**20’**

**Mean SpO_2_ (SD)**	96.8 (2.1)	96.4 (2.2)	96.38 (2.1)	96.33 (2.18)	96.24 (2.3)	96.3 (2.3)	96.3 (2.1)	96.2 (2.4)	96.3 (2.1)	96.4 (2.1)
**Mean SaO_2_-SpO_2_ (SD)**	−1.14 (1.32)	−0.71 (1.5)	−0.7 (1.49)	−0.68 (1.6)	−0.59 (1.69)	−0.61 (1.72)	−0.68 (1.46)	−0.52 (2)	−0.61 (1.45)	−0.78 (1.48)
**95 % CI**	−0.86; −1.42	−0.4; −1.02	−0.38; −1.01	−0.34; −1.01	−0.23; −0.94	−0.24; −0.97	−0.37; −0.99	−0.09; −0.94	−0.3; −0.9	−0.46; −1.09
**Pearson correlation coefficients (*r*)**	0.822	0.782	0.772	0.738	0.721	0.710	0.780	0.634	0.788	0.776
**ICC**	0.818	0.780	0.768	0.735	0.721	0.709	0.775	0.633	0.785	0.772

The upper row shows the time in minutes. SpO_2_: pulsoximeter oxygen saturation. SD: standard deviation. CI: confidence interval. ICC: intraclass correlation coefficient for individual measures

**Table 4.** Mean baseline PaCO_2_ and transcutaneous CO_2_ readings every 2 minutes (in mm Hg) in patients with and without several degrees of overshoot.

**Overshoot**	**PaCO_2_ (mmHg)**	**2’**	**4’**	**6’**	**8’**	**10’**	**12’**	**14’**	**16’**	**18’**	**20’**

**None (n = 29)**	40.2 ± 4.9	26.3 ± 8.7	32.7 ± 8.2	35.2 ± 7.1	36.2 ± 6.4	36.9 ± 5.7	37.1 ± 5.5	37.3 ± 5.6	38 ± 5.6	37.6 ± 5.5	38 ± 5
**Light (n = 33)**	39.5 ± 4.3	30.9 ± 6.3	36.3 ± 5	38.4 ± 4.8	39.1 ± 4.9	39.2 ± 4.8	39 ± 4.7	38.7 ± 4.7	38.5 ± 4.7	38.6 ± 4.7	38.2 ± 4.6
**Moderate (n = 20)**	39.9 ± 6.1	31.5 ± 5.3	38.0 ± 5.6^b^	40.7 ± 6.3 ^b^	41.6 ± 6.6 ^b^	41.7 ± 6.9^b^	41 ± 7	40.0 ± 6	39.5 ± 6.5	39 ± 6.4	39 ± 6.2
**Severe (n = 9)**	40.2 ± 3.6	36.3 ± 6.5^a^	41.3 ± 4.8^b^	43.3 ± 4.6 ^b^	43.5 ± 5 ^b^	42.9 ± 4.2 ^b^	40.7 ± 3.7	39.5 ± 3.4	38.4 ± 3.5	38.4 ± 3.9	39.5 ± 4.1

The upper row shows the values of arterial PCO_2_ and the time in minutes.

a) p < 0.05 between severe overshoot and no overshoot (ANOVA test, Bonferroni *post hoc* analysis).

b) p < 0.05 between no overshoot vs. moderate and severe overshoot.

## Abbreviations:

SpO_2_: oxygen saturation
TcPCO_2_: transcutaneous partial pressure of carbon dioxide
PaCO_2_: arterial pressure of carbon dioxide
PaO_2_: arterial oxygen pressure
ABG: arterial blood gas analysis
SpO_2_: transcutaneous arterial oxygen saturation
FEV_1_: forced spirometry value in the first second
FVC: forced vital capacity
PFTs: pulmonary function testings
